# Chemical Content and Cytotoxic Activity on Various Cancer Cell Lines of Chaga (*Inonotus obliquus*) Growing on *Betula pendula* and *Betula pubescens*

**DOI:** 10.3390/ph17081013

**Published:** 2024-08-01

**Authors:** Ain Raal, Hedi Kaldmäe, Karin Kütt, Katrin Jürimaa, Maidu Silm, Uko Bleive, Alar Aluvee, Kalev Adamson, Marili Vester, Mart Erik, Oleh Koshovyi, Khan Viet Nguyen, Hoai Thi Nguyen, Rein Drenkhan

**Affiliations:** 1 Institute of Pharmacy, Faculty of Medicine, University of Tartu, Nooruse 1, 50411 Tartu, Estonia; oleh.koshovyi@ut.ee; 2 Polli Horticultural Research Centre, Chair of Horticulture, Institute of Agricultural and Environmental Sciences, Estonian University of Life Sciences, Uus 2, Polli, 69108 Mulgi Parish, Estonia; hedi.kaldmae@emu.ee (H.K.); uko.bleive@emu.ee (U.B.); alar.aluvee@emu.ee (A.A.); 3 Institute of Forestry and Engineering, Chair of Silviculture and Forest Ecology, Estonian University of Life Sciences, Kreutzwaldi 5, 51006 Tartu, Estonia; karin.kytt@emu.ee (K.K.); katrin.jyrimaa@emu.ee (K.J.); kalev.adamson@emu.ee (K.A.); marili.vester@emu.ee (M.V.); rein.drenkhan@emu.ee (R.D.); 4 Institute of Agricultural and Environmental Sciences, Estonian University of Life Sciences, Kreutzwaldi 5, 51006 Tartu, Estonia; maidu.silm@emu.ee; 5 Inopure OÜ, 51010 Tartu, Estonia; mart53@gmail.com; 6 Faculty of Pharmacy, Hue University of Medicine and Pharmacy, Hue University, 06 Ngo Quyen, Hue City 530000, Vietnam; nvkhan@hueuni.edu.vn (K.V.N.); nthoai@hueuni.edu.vn (H.T.N.)

**Keywords:** silver birch, downy birch, Estonia, glucans, triterpenoids, inotodiol, betulin, betulinic acid

## Abstract

Chaga mushroom (*Inonotus obliquus*) is a pathogenic fungus that grows mostly on birch species (*Betula pendula* Roth and *B. pubescens* Ehrh.) and has traditionally been used as an anticancer medicine. This study aimed to compare the chemical composition and cytotoxic activity of chagas growing on both *Betula* spp. on various cancer cell lines. The freeze-dried extracts contained triterpenes inotodiol, lanosterol betulin, and betulinic acid typical to conks growing on *Betula* species. The cytotoxic activity of chaga growing on *Betula pendula* and *B. pubescens* 80% ethanolic extracts against 31 human cancer cell lines was evaluated by a sulforhodamine B assay. Chaga extract showed moderate activity against all cancer cell lines examined; it did not result in high cytotoxicity (IC_50_ ≤ 20 µg/mL). The strongest inhibitions were observed with chaga (growing on *B. pendula*) extract on the HepG2 and CAL-62 cell line and with chaga (from *B. pubescens*) extract on the HepG2 cell line, with IC_50_ values of 37.71, 43.30, and 49.99 μg/mL, respectively. The chaga extracts from *B. pendula* exert somewhat stronger effects on most cancer cell lines studied than *B. pubescens* extracts, which can be attributed to a higher content of inotodiol in *B. pendula* extracts. This study highlights the potential of chaga as a source of bioactive compounds with selective anticancer properties. To the best of our knowledge, this study is the first investigation of the chemical composition of *I. obliquus* parasitizing on *B. pubescens*.

## 1. Introduction

A basidiomycete fungus chaga (*Inonotus obliquus*) typically occurs on the trunks of trees growing in the Northern Hemisphere [1]. This pathogenic fungus grows mostly on birch species (*Betula* spp.) and is less known on other trees [2,3]. As a real parasite, chaga kills the host organism to fulfil its lifecycle [4]. Due to being found mainly on birch trunks, the chaga drug is known in pharmacy as *Fungus betulinus*, not to be confused with another species, *Piptoporus betulinus.*

Chaga contains triterpenes, steroids, alkaloid-like compounds, organic and phenolic acids (oxalic, vanillic, cinnamic, gallic, protocatechuic, *p*-hydroxybenzoic acids, etc), polysaccharides [5,6], simple phenols, flavonoids, iridoids, nonsaturated fatty acids, vitamin K, coenzyme Q, phospholipids, and glycolipids [7], isocoumarins, diarylheptanoids [8], coumarins, quinones, and styrylpyrones, as well as vitamins and minerals [9].

As a natural source of biologically active substances, the chaga has been traditionally used as an antitumour, anti-inflammatory, antibacterial, hepatoprotective, and antioxidant natural remedy [10]. Chaga has been recognised for its medicinal uses since the sixteenth century [11]. Owing to containing a range of pharmaceutical and nutraceutical value, including polyphenols, triterpenoids, polysaccharides, and lignin, chaga has demonstrated diverse therapeutic effects. These encompass antioxidant, anti-inflammatory, antibacterial, antiviral, hepatoprotective, antidiabetic, anti-obesity, renoprotective, hepatoprotective, immunomodulatory, antitumour, and anti-fatigue activities. Significantly, chaga has shown efficacy in anticancer activities [11,12,13]. The conks of *I. obliquus* have been the best-known and most popular anticancer remedy in Estonian ethnomedicine among other 43 natural drugs [14]. Nowadays, chaga is rather well studied and primarily known as a promising natural material against cancer and immunotherapy [6,9,15,16,17]. The anticancer activity of different chaga extracts has been studied by several researchers [15,18,19,20,21,22,23,24]. The water extract of chaga exhibited a potential anticancer activity against B16-F10 melanoma cells in vitro and in vivo through the inhibition of proliferation and induction of differentiation and apoptosis of cancer cells [25]. Szychowski et al. (2018) compared the 80% ethanol and methanol extracts and showed their different biological activities. The ethanol extract exhibited the highest properties inhibiting the activity of xanthine oxidase and radical scavenging activities [24].

Most studies have been conducted on cell lines from the digestive system [26]. For example, a hot-water chaga extract inhibited the proliferation of human colorectal cancer cells (HT-29) [27]. Lee et al. (2015) showed that 95% ethanol extract of chaga is active in HT-29 human colon cancer cells [28].

Several studies of the chaga extracts showed excellent anticancer activities against colon cancers, but the bioactive compounds exhibiting those effects remain unknown [27]. The water-soluble polysaccharide fraction of chaga is a promising group of substances associated with anticancer activity and insulin-improving sensitivity [29,30]. Thus, the high content of polysaccharides seems to be responsible for the anticancer potential of chaga [31]. Conversely, anticancer potential has been found in all types of extracts made by different solvents [32]. Several studies [16,33,34,35] have shown the cytotoxic activity of chaga triterpenoids against various cancer cells soluble in 80% ethanol [4].

The aim of the study was to compare the (1) chemical composition and (2) cytotoxic activity of chagas growing on *Betula pendula* and *B. pubescens* on various cancer cell lines.

To the best of our knowledge, except for cytotoxic activity against HepG2 cell lines, this is the first investigation of cytotoxicities against the tested cell lines of chaga parasitizing on *B. pendula* Roth and *B. pubescens* Ehrh.

## 2. Results

### 2.1. Phytochemical Study of Chaga

#### Extracts and Content of Biochemical Compounds

Freeze-dried ethanol extracts of chaga growing on two species, *Betula pendula* and *B. pubescens*, were prepared and analysed for triterpenoid composition and antioxidant activity (Table 1).

Although the ratio of dried raw material and solvent was the same for both samples, the yield of freeze-dried dry extract from samples differed slightly: 50 g of *B. pendula* conks resulted in 1.26 g of dry extract and the same amount of *B. pubescens* gave 1.45 g of dry extract.

There were no significant differences in the content of sitosterol and betulinic acid. Also, antioxidant activity evaluated using the DPPH method did not reveal any differences between these extracts (Table 1). Significant differences were revealed between the content of lanosterol, inotodiol, and betulin in the extracts from conks from *B. pubescens* and *B. pendula*. The content of inotodiol in these samples was negatively correlated with the content of betulin and lanosterol, while betulin and lanosterol content had a strong positive correlation.

### 2.2. Cytotoxic Activity of Chaga Extracts

The cytotoxic activity of chaga (growing on *B. pendula* and *B. pubescens*) extract against thirty-one human cancer cell lines (Table 2) was evaluated by a sulforhodamine B assay [36,37]. The results of the cytotoxic assays are summarised in Table 3. These data showed that the strongest inhibitions were observed with chaga (growing on *B. pendula*) extract on the HepG2 and CAL-62 cell lines and with chaga (growing on *B. pubescens*) extract on HepG2 cell line, with IC_50_ values of 37.71, 43.30, and 49.99 μg/mL, respectively. Generally, chaga extracts demonstrated moderate activity against all cancer cell lines examined; high cytotoxicity (IC50 ≤ 20 µg/mL) was not detected. The extracts from *B. pendula* showed somewhat stronger experimental results than *B. pubescens* extracts on most cancer cell lines studied.

## 3. Discussion

The most abundant of the identified triterpenes was inotodiol, as has been reported in earlier studies [4,38]. The content of lanosterol, inotodiol, and betulin significantly varied among the extracts from *B. pubescens* and *B. pendula*. Inotodiol was higher in *B. pendula* extract, and lanosterol and betulinic acid content in *B. pubescens* extracts. Correlation analysis revealed a positive correlation between inotodiol content in the extract and the effect on growth inhibition on most of the tested cell lines. A negative correlation was revealed between inotodiol and growth inhibition only on cell lines NTERA-2 and 8505c.

Also, in a previous study [4], the concentration of inotodiol was much higher than other triterpenes lanosterol, betulin, and betulinic acid. Upska et al. studied the extractability of chaga active ingredients using different methods and both polar and non-polar solvents. The best extractability of inotodiol was obtained by Soxhlet extraction using cyclohexane [39]. Inotodiol and lanosterol are important compounds in chaga’s anticancer effects [40,41]. Inotodiol and other lanostanes were effective against A549 cancer cells [42,43]. Inotodiol and lanosterol isolated from the conks of *Betula* spp. have immunological effects [17,44]. Inotodiol and lanosterol may also have cosmetic importance as they activate tyrosinase and increase pigment synthesis in skin cells [45]. Tian et al. showed that triterpenes of chaga may be an effective natural aid for treating and protecting various kidney diseases [46].

The antioxidant activity measurements performed using a DPPH assay showed the same results for chaga from both *Betula* spp. The level of antioxidant activity may depend on the solvent used to extract chaga and the origin of the raw material. The difference in the DPPH scavenging activity may be up to 40 times different in chagas from various countries [6]. The scavenging activity of the water and ethanol extracts of chaga conks showed a similar level, but the methanol extract had maximum IC_50_ values of 18.96, 16.25, and 24.90 mg/mL, respectively [47].

The main finding was that the chaga extracts showed moderate activity against all cancer cell lines examined (IC_50_ < 21 µg/mL). An earlier study [15] with methanol extract of chaga collected from the herb farm Kubja Ürditalu, Estonia, N59.054344, E25.963234, and the commercial sample purchased from a retail pharmacy in Tartu, Estonia, revealed potent cytotoxic effects against promyelocytic leukaemia and lung adenocarcinoma cells, with IC_50_ values of 32.2 and 38.0 µg/mL, respectively. Moreover, the extract showed weak cytotoxicity (41.3–57.7 µg/mL) against cells from colon adenocarcinoma, liver hepatocellular carcinoma, oral epidermoid carcinoma, and prostate cancer [15]. This study assessed the anticancer properties of chaga (growing on *B. pendula* and *B. pubescens*) extracts across a diverse range of cancer cell lines. The results demonstrated a broad spectrum of cytotoxic effects against 24 cancer cell lines for chaga (growing on *B. pendula*) extract, i.e., KB, MCF-7, LNCaP, A549, LLC, HepG2, Hep3B, HL-60, MKN7, SW626, Hela, SW480, RD, SK-Mel-2, OCI/AML3, K562, Jurkat, CAL-62, T24, Huh-7, HT29, 8505c, SNU1, and MDA-MB-231, and against 17 cancer cell lines for chaga (growing on *B. pubescens*) extract, i.e., KB, MCF-7, CL141, A549, LLC, HepG2, HL-60, SNU1, Hela, RD, SK-Mel-2, K562, Jurkat, 8505c, CAL-62, T24, and NTERA-2. Both chagas growing on these two host species possessed the most potential cytotoxic activities against the HepG2 cell line, with IC_50_ values of 37.71 and 49.99 μg/mL, respectively. Furthermore, the extract of chaga (growing on *B. pendula*) exhibited remarkable cytotoxicity against the CAL-62 cell line, with an IC_50_ value of 43.30 μg/mL. Notably, liver cancer in men and thyroid cancer in women rank among the five most common types of cancer [23].

Here are some comparative data obtained using the same method in other natural products, which help to orient the strength of chaga’s cytotoxic activity. The strongest anticancer effect of *Matricaria chamomilla* methanol extract against SK-MEL-2 cells was IC_50_ 40.7 μg/mL, but the extract of *Calendula officinalis* showed no remarkable cytotoxic activity against SK-MEL-2 and KB cells (IC_50_ 62.6 and 79.2 μg/mL, respectively) [48]. The essential oils of *Anthemis sylvestris* roots and aerial parts had the strongest anticancer activity on KB cells (IC_50_ 19.7 μg/mL and 19.8 μg/mL, respectively), while the methanolic extract had no effect [37]. The *Pinus sylvestris* needle methanol extract suppresses the viability of MDA-MB-231 cells on the level IC_50_ 35 μg/mL [49]. In this context of anticancer activity, the effect of chaga’s extract, having the strongest values between 37.7 and 43.3 μg/mL, showed remarkable but moderate effects.

Chaga has received considerable attention from many researchers due to its numerous biological attributes, particularly its potential anticancer effects. According to Ma et al., the petroleum ether and ethyl acetate extracts of chaga displayed significant cytotoxic activities against the human prostatic carcinoma cell PC_3_ and breast carcinoma cell MDA-MB-231 [18]. The inhibitory effects were mainly attributed to ergosterol peroxide and trametenolic acid [18]. The aqueous extract of chaga in France containing a high content of betulin and betulinic acid and inotodiol showed an effect on human lung adenocarcinoma cells [21]. Noticeably, chaga extract was reported to markedly reduce HepG2 cell viability due to G0/G1-phase arrest and apoptotic cell death, thus leading to downregulation of p53, pRb, p27, cyclins D1, D2, E, cyclin-dependent kinase (Cdk) 2, Cdk4, and Cdk6 expression [50]. Through Matrigel-coated filters, the methanolic extract and its EtOAc-soluble fraction from Chaga collected in Japan showed the significant invasion inhibition of human fibrosarcoma HT 1080 cells. Importantly, compound 3*β*-hydroxylanosta-8,24-dien-21-al exhibited a strong inhibitory effect on HT 1080 cells. Furthermore, the methanolic extract of this sample significantly suppressed the formation of lung tumours in mice at 500 mg/kg/d [20]. The methanolic extract of Russian chaga purchased in Korea showed inhibitory effects toward four human lung adenocarcinoma cell lines (including A549, H1264, H1299, and Calu-6) through induction of apoptosis accompanied by caspase-3 cleavage. Significantly, compounds 3*β*-hydroxylanosta-8,24-dien-21-al, trametenolic acid, and 3*β*-hydroxy-5*α*lanosta-8,25-dien-21-oic acid isolated from this extract showed cytotoxicities against these cell lines, with IC_50_ values ranging from 75.1 to 227.4 μM in the same pattern [51]. The aqueous extract of chaga (in Korea) displayed anti-proliferative activity on HCT116 and DLDl cell lines and reduced intestinal polyps in APC^Min/+^ and colon tumours in AOM/DSS-treated mice through downregulation of Wnt/β-catenin and NF-κB pathways [52]. The extract of chaga collected from birch trees in Japan successfully reduced tumours in both tumour-bearing mice and metastatic mice by promoting energy metabolism [19]. Additionally, the administration of chaga obtained in Japan could induce necrotic lesions, resulting in a decrease in the growth of the tumour and the weight of dog bladder cancer organoid-derived xenografts [22].

A limited number of fungi have been studied for bioactive compounds that can help treat various diseases including anticancer activity. Promising fungal species’ anticancer activity on human cancer cell lines are many, but numerous studies, including clinical studies, are focused on *Lentinula edodes*, *Coriolus versicolor*, and *Ganoderma lucidum* [53]. For example, Ganoderma species have demonstrated a wide range of health benefits, such as anticancer, anti-immunomodulatory, anti-inflammatory, antimicrobial, and antioxidant effects, which can be attributed to their bioactive compounds [54]. Also, *Lentinula edoides* has shown antitumour, cytotoxic, and immunomodulating moiety in treating various cancers [55]. Related to these named fungi, most in vitro cell studies use breast cancer cell lines (43.9%), followed by lung (14%) and colorectal cancer cell lines (13.1%) [53].

The current work and some others [4,15,56] indicate that fungal species origin and the fungus host or growth substrate affect the source of useful bioactive compounds. *Inonotus obliquus* grows naturally on *Betula* spp. and on *Alnus* spp. as well as some other broadleaved trees in northern Baltic conditions [2,3]. In this area, the fungus grows on *B. pendula* and *B. pubescens* [2,3,57], and *B. pendula* is a widely known host to the chaga [4], but not *B. pubescens*. However, nothing is known about the bioactive compounds of the chaga growing on *B. pubescens*. The current work shows evidently that the bioactive compounds of chaga differ significantly according to the origin of quite close host species such as *B. pendula* and *B. pubescens*. This indicates that the fungus origin and background are important for further analyses including effects on different human cancer cell lines.

These findings highlight the potential of chaga as a source of bioactive compounds with selective anticancer properties only on the HepG2 cell line, encouraging further exploration of its therapeutic mechanisms and potential applications in cancer treatment.

## 4. Materials and Methods

### 4.1. Sample Sites, Fungal Isolation, Detection, and Preparation

The conks of *I. obliquus* were collected from *B. pendula* and *B. pubescens,* originating from Estonia between 21 December 2019 and 3 February 2022 (Table 4). The isolates were obtained from fresh conks to grow on 2% malt-extract agar (Biolife, Milano, Italy) plates.

The DNA of *I. obliquus* isolates was extracted using a GeneJET Genomic DNA Purification Kit (Thermo Scientific, Vilnius, Lithuania), and the fungus was detected as described by Drenkhan et al. [58]. ITS-PCR products from the isolates were sequenced at the Estonian Biocentre in Tartu. The ITS sequences were edited using the BioEdit program, Version 7.2.5 [59], and deposited in a GenBank (see Table 4). BLAST searches for the fungal taxa confirmation were performed in the GenBank database (NCBI). All the pure cultures were deposited into the Fungal Culture Collection (TFC) and GenBank (NCBI).

Before extractions and biochemical analysis, the conks were kept in a freezer at −20 °C. Then, the collected conks were dried in a laboratory oven (MMM Medcenter Einrichtungen GmbH, Planegg, Germany) at 50 °C, and all the material was ground into a coarse powder using a cutting mill Retch SM 300 with a 1 mm sieve (Retsch, Haan, Germany). Extracts were prepared from the mix of 10 individual conks samples per host species in equal proportions (see Table 4). For extract preparation, 500 mL of 80% ethanol was added to 50 g of dried and milled sample and agitated at room temperature for 12 h using Biosan ES20 orbital shaker. After vacuum filtration, the extracts were concentrated on rotary evaporator RV 10 control Flex (IKA-Werke GmbH & Co. KG., Staufen im Breisgau, Germany) to remove most of the ethanol and further dried in the VirTis AdVantage 2.0 EL freeze-drier (SP Scientific, Santa Cruz, CA, USA). Bioactive compound detection from *I. obliquus* was performed according to [4]. We selected 80% ethanol for extraction based on the pre-trial from our previous research [4], where we optimised the sample preparation to determine free radical scavenging activity, polyphenols, and triterpenes content. In the pre-trial, we compared the following solvents: ethyl acetate, hexane, 80% EtOH, and a mixture of dichloromethane with MetOH and water in a proportion of 5:3:2.

Antioxidant activity measurements were performed in triplicate using a 2.2-diphenyl-picrylhydrazyl (DPPH) assay [60]. The absorbance values of the samples were measured at 515 nm using a spectrophotometer (UV-1800, Shimadzu, Kyoto, Japan). The results were expressed in mg of gallic acid equivalent per g of dry weight (mg GA eq./g).

**Figure 1:**
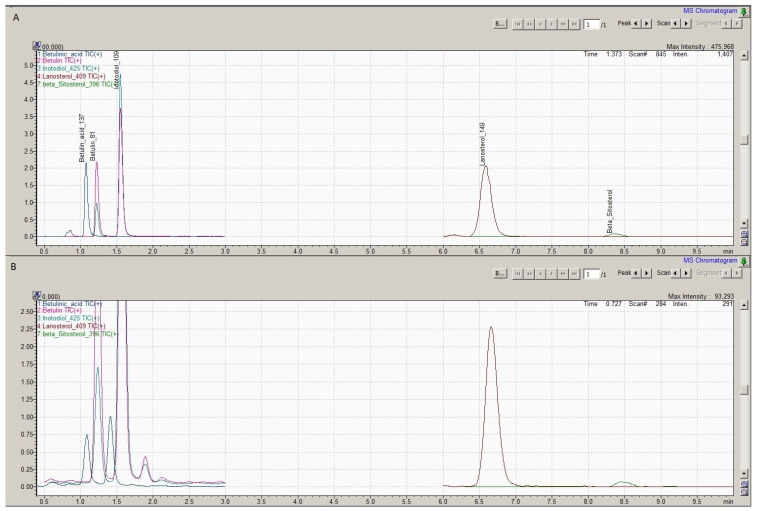
Total ion chromatogram (**A**) of the solution containing the standard compounds: betulinic acid, betulin, inotodiol, lanosterol, and beta-sitosterol and total ion chromatogram of *Betula pubescence* extract (**B**).

Qualitative and quantitative analyses were performed on a Shimadzu Nexera X2 UHPLC with mass spectrometer LCMS 8040 (Shimadzu Scientific Instruments, Kyoto, Japan). The UHPLC system had a binary solvent delivery pump LC-30AD, an autosampler Sil-30AC, a column oven CTO-20AC, and a diode array detector SPD-M20A. Five triterpenoids (betulinic acid, betulin, lanosterol, inotodiol, and sitosterol) were identified by comparing the retention times and parent and daughter ion masses with those of the standard compounds (Figure 1). Chromatographic separation was performed using the Ascentis Express column (C30 50 × 4.6 mm, Merck, Darmstadt, Germany) at 40 °C. The flow rate of the mobile phase was 1 mL/min, and the injected sample size was 1 µL. Mobile phases consisted of 1% formic acid in Milli-Q water (mobile phase A) and 1% formic acid in acetonitrile (mobile phase B). Separation was performed for 10 min at isocratic conditions with 92% of mobile phase B and 8% of mobile phase A. All samples were kept at 4 °C during the analysis. MS data acquisitions were performed on LCMS 8040 with the APCI source. All samples were analysed in triplicate, and the results were expressed as mg per g of dry weight (mg/g).

The standards betulin, betulinic acid, and lanosterol were purchased from Cayman Chemical Company (Ann Arbor, MI, USA), and inotodiol was from Aobious (Gloucester, MA, USA). All other standards (sitosterol, gallic acid) and chemicals (formic acid, methanol) used were of analytical grade and purchased from Sigma (Darmstadt, Germany).

The dry matter content in the samples was determined at 105 °C using the moisture analyser Precisa EM 120 HR (Precisa Gravimetrics AG, Dietikon, Switzerland).

### 4.2. Cytotoxic Assay

A total of 31 human cancer cell lines (Table 1 and Table 2) were cultivated at 37 °C in a humidified atmosphere containing 5% carbon dioxide. An SRB cytotoxic assay for monolayer cells was analysed according to the method described in [61,62].

The 80% ethanolic dry extracts were first stocked in dimethyl sulfoxide (DMSO) 100% at the concentration of 20 mg/mL. The samples were then prepared in a range of diluted concentrations as 2000–400–80–16 µg/mL using basic RPMI medium (*w*/*o* FBS) in 96-well sample plates. Then, 10 µL from each well (with the diluted sample above) was added to the tested cell pre-seeded (190 µL) well to evaluate cytotoxic activities. Thus, the final concentration of the sample was 100–20–4–0.8 µg/mL. Ellipticine was tested with the final concentration of 20–4–0.8–0.16 µg/mL.

The effects of *Betula* chaga hydroethanolic extracts on the viability of malignant cells were determined by sulforhodamine B cytotoxic assay [61,62]. Briefly, cells were grown in 96-well microtiter plates, each containing 190 μL of medium. After 24 h, 10 μL of test samples dissolved in DMSO were added to each well. One plate with no samples served as a day 0 control. The cells were continuously cultured for an additional 48 h, fixed with trichloroacetic acid, and stained with sulforhodamine B, followed by determining optical densities at 515 nm using a Microplate Reader (BioRad, Hercules, CA, USA). The percentage of growth inhibition was calculated using the following equation:

[OD (reagent)–OD (day 0)] ×100% Growth = [OD (negative control DMSO 10%)–OD (day 0)]

where OD is the optical density or absorbance value. The potent anticancer agent ellipticine was used as a positive control.

Based on the U.S. National Cancer Institute, IC_50_ ≤ 20 µg/mL = highly cytotoxic, 21 ≤ IC_50_ ≤ 200 µg/mL = moderately cytotoxic, 201 ≤ IC_50_ ≤ 500 µg/mL = weakly cytotoxic, and IC_50_ > 500 µg/mL = not cytotoxicity [63,64].

### 4.3. Statistics

Biochemical analysis data were analysed using Jamovi software [65]. Metabolite concentrations were analysed by one-way analysis of variance (ANOVA). Differences between means were assessed with the post hoc Tukey test. The IC_50_ values were analysed using TableCurve 2Dv4 software.

## 5. Conclusions

The strongest inhibitions were observed on the HepG2 cell line with chaga grown on both hosts (*Betula pendula* and *B. pubescens*). In general, chaga extract showed moderate activity against all 31 cancer cell lines studied.

The *B. pendula* origin chaga extracts exert somewhat stronger effects on most cancer cell lines studied than *B. pubescens* parasitizing chaga extracts. This effect can be attributed to a higher content of inotodiol in *B. pendula* origin extracts compared to *B. pubescens*. This is also the first comparative investigation of the chemical composition of *I. obliquus* parasitizing on *B. pendula* and *B. pubescens*.

The results of the study highlight the potential of chaga as a source of bioactive compounds with selective anticancer properties, encouraging further exploration of its therapeutic mechanisms and potential applications in cancer treatment.

## Figures and Tables

**Table 1:** The average content of triterpenoids and the antioxidant activity of freeze-dried extracts of chaga conks growing on *Betula pendula* and *B. pubescens*.

Antioxidant Activity	Triterpenoids					Species
Aox GA eq ^1^ mg/g	Sitosterol mg/g	Lanosterol mg/g	Betulin mg/g	Betulinic Acid mg/g	Inotodiol mg/g	
21.5 ± 0.065	0.780 ± 0.149	8.89 ± 0.497 ^a^	7.22 ± 0.104 ^a^	2.77 ± 0.165	169 ± 6.60 ^a^	*B. pendula*
						dry extract
21.4 ± 0.045	0.770 ± 0.0964	11.1 ± 0.364 ^ab^	14.9 ± 0.690 ^b^	2.05 ± 0.476	149 ± 2.30 ^b^	*B. pubescens* dry extract

All values are given per g of freeze-dried extract. All values are means ± standard deviation (n = 3); mean values within a column marked with different letters ^a,b^ are significantly different at *p* < 0.05. Differences between means were assessed with ANOVA and post hoc Tukey test. ^1^ Aox GA eq. mg/g antioxidant activity expressed as gallic acid equivalent mg/g.

**Table 2:** Abbreviations of 31 human cancerous cell lines.

Basic Characteristics	Name
Human carcinoma in the mouth	KB
Human breast adenocarcinoma	MDA-MB-231
Human breast carcinoma	MCF7
Human prostate carcinoma	LNCaP
Human lung carcinoma	SK-LU-1
Human lung adenocarcinoma	CL141
Human lung carcinoma	A549
Lewis lung carcinoma–high metastasis	LLC
Human hepatocellular carcinoma	HepG2
Human hepatocellular carcinoma	Hep3B
Human hepatocyte-derived carcinoma	Huh7
Drug-resistant human hepatocyte-derived carcinoma	Huh 7R
Human acute leukaemia	HL-60
Human differentiated human gastric adenocarcinoma	MKN7
Human gastric carcinoma	NCI-N87
Human gastric adenocarcinoma	AGS
Human stomach carcinoma	SNU-1
Human ovarian adenocarcinoma	SW626
Human cervix carcinoma	Hela
Human colon adenocarcinoma	SW480
Human colorectal adenocarcinoma	HT-29
Human rhabdomyosarcoma	RD
Human malignant melanoma	SK-Mel-2
Human kidney adenocarcinoma	ACHN
Human acute myeloid leukaemia	OCI/AML3
Human chronic myelogenous leukaemia	K562
Human acute T cell leukaemia	Jurkat
Human undifferentiated thyroid carcinoma	8505c
Human thyroid anaplastic carcinoma	CAL-62
Human urine bladder carcinoma	T24
Pluripotent human embryonal carcinoma	NTERA2

**Table 3:** Cytotoxic activity of chaga extracts on various cancer cell lines.

Cancer Cell Line	IC_50_^a^ (µg/mL)		
	*Betula pendula*	*Betula pubescens*	Ellipticine ^b^
KB	63.35 ± 2.89	74.90 ± 1.38	0.49 ± 0.03
MCF-7	77.92 ± 4.49	90.87 ± 3.82	0.61 ± 0.05
MDA-MB-231	97.73 ± 3.78	>100	0.68 ± 0.07
LNCaP	70.46 ± 2.17	>100	0.44 ± 0.04
SK-LU-1	>100	>100	0.45 ± 0.05
CL141	>100	92.81 ± 4.55	0.55 ± 0.03
A549	85.44 ± 5.27	78.32 ± 3.31	0.62 ± 0.04
LLC	52.29 ± 3.57	55.89 ± 2.37	0.35 ± 0.03
HepG2	37.71 ± 2.08	49.99 ± 1.94	0.37 ± 0.02
Hep3B	76.70 ± 3.42	>100	0.73 ± 0.04
Huh-7	95.34 ± 3.36	>100	0.56 ± 0.05
Huh-7R	>100	>100	0.66 ± 0.06
HL-60	79.02 ± 2.82	71.21 ± 2.41	0.64 ± 0.05
MKN7	68.85 ± 4.06	>100	0.36 ± 0.02
NCI-N87	>100	>100	0.75 ± 0.06
AGS	>100	>100	0.68 ± 0.03
SNU1	95.82 ± 2.97	97.02 ± 4.61	0.44 ± 0.02
SW626	82.88 ± 5.84	>100	0.51 ± 0.03
Hela	68.50 ± 3.45	94.28 ± 4.69	0.50 ± 0.04
SW480	87.10 ± 5.19	>100	0.53 ± 0.02
HT29	86.08 ± 6.03	>100	0.37 ± 0.03
RD	84.12 ± 5.95	82.08 ± 3.96	0.74 ± 0.02
SK-Mel-2	62.82 ± 3.20	71.18 ± 4.61	0.57 ± 0.02
ACHN	>100	>100	0.77 ± 0.02
OCI/AML3	55.57 ± 4.33	>100	0.57 ± 0.03
K562	61.41 ± 3.28	75.00 ± 3.17	0.46 ± 0.04
Jurkat	63.48 ± 3.15	76.16 ± 2.88	0.68 ± 0.05
8505c	91.50 ± 1.80	69.42 ± 3.10	0.59 ± 0.02
CAL-62	43.30 ± 2.52	69.42 ± 3.10	0.59 ± 0.02
T24	76.76 ± 4.71	93.81 ± 5.66	0.54 ± 0.02
NTERA-2	>100	70.11 ± 3.93	0.57 ± 0.03

^a^ IC_50_ (concentration that inhibits 50% of cell growth). ^b^ Positive control.

**Table 4:** Origin and hosts of *Inonotus obliquus* isolated from the conks and used in this study’s fungal strains.

Accession No. in GenBank **	Fungal Collection Code *	Sampling Date	Geographical Coordinates	Host	Strain No.
OP019325	TFC101258	21 December 2019	N58.90373, E26.44672	*Betula pendula*	PAT29045
OP942253	TFC101271	30 December 2019	N58.06711, E26.42738	*Betula pendula*	PAT29055
PP346417	TFC101304	11 February 2020	N58.2567, E26.6659	*Betula pendula*	PAT29051
OP942256	TFC101274	9 November 2021	N58.52718, E22.91413	*Betula pendula*	PATKA880
OP942259	TFC101277	11 November 2021	N58.91147, E22.35235	*Betula pendula*	PATKA896
OP942263	TFC101281	27 January 2022	N57.82154, E27.48639	*Betula pendula*	PATKA1567
OP942264	TFC101282	27 January 2022	N57.82154, E27.48639	*Betula pendula*	PATKA1568
OP942268	TFC101286	25 January 2022	N59.01350, E27.60455	*Betula pendula*	PATKA1575
OP942272	TFC101290	3 February 2022	N59.27381, E25.36868	*Betula pendula*	PATKA1679
OP942273	TFC101291	3 February 2022	N59.27381, E25.36868	*Betula pendula*	PATKA1680
OP942269	TFC101287	25 January 2022	N59.01654, E27.44207	*Betula pubescens*	PATKA1576
OP942270	TFC101288	25 January 2022	N59.01367, E27.53098	*Betula pubescens*	PATKA1577
OP942260	TFC101278	11 November 2021	N58.98419, E22.72165	*Betula pubescens*	PATKA900
PP346418	TFC101305	17 October 2020	N58.9065, E26.0808	*Betula pubescens*	PATRD3354
PP346419	TFC101306	29 October 2020	N58.3203, E25.7080	*Betula pubescens*	PATRD3364
PP346420	TFC101307	5 March 2021	N58.3776, E25.9267	*Betula pubescens*	PATRD3402
-	TFC101308	15 March 2021	N58.9566, E25.4320	*Betula pubescens*	PATRD3355_1
PP346421	TFC101309	27 October 2021	N58.6825, E25.6854	*Betula pubescens*	PATRD3356
PP346422	TFC101310	17 December 2021	N59.4096, E26.6767	*Betula pubescens*	PATRD3386
PP346423	TFC101311	17 December 2021	N59.4932, E26.5887	*Betula pubescens*	PATRD3385

* Tartu Fungal Collection in Estonian University of Life Sciences, Estonia (TFC). ** ITS sequences in GenBank (NCBI).

## Data Availability

Further inquiries can be directed to the corresponding author.
